# Genome-Wide Analysis of Cold Adaptation in Indigenous Siberian Populations

**DOI:** 10.1371/journal.pone.0098076

**Published:** 2014-05-21

**Authors:** Alexia Cardona, Luca Pagani, Tiago Antao, Daniel J. Lawson, Christina A. Eichstaedt, Bryndis Yngvadottir, Ma Than Than Shwe, Joseph Wee, Irene Gallego Romero, Srilakshmi Raj, Mait Metspalu, Richard Villems, Eske Willerslev, Chris Tyler-Smith, Boris A. Malyarchuk, Miroslava V. Derenko, Toomas Kivisild

**Affiliations:** 1 Department of Archaeology and Anthropology, University of Cambridge, Cambridge, United Kingdom; 2 Wellcome Trust Sanger Institute, Hinxton, United Kingdom; 3 The Wellcome Trust Centre for Human Genetics, University of Oxford, Oxford, United Kingdom; 4 Department of Mathematics, University of Bristol, Bristol, United Kingdom; 5 National Cancer Centre Singapore, Singapore, Singapore; 6 Department of Human Genetics, University of Chicago, Chicago, Illinois, United States of America; 7 Department of Molecular Biology and Genetics, Cornell University, Ithaca, New York, United States of America; 8 Department of Evolutionary Biology, Institute of Molecular and Cell Biology, University of Tartu, Tartu, Estonia; 9 Estonian Biocentre, Tartu, Estonia; 10 Centre for GeoGenetics, Natural History Museum of Denmark, University of Copenhagen, Copenhagen, Denmark; 11 Institute of Biological Problems of the North, Russian Academy of Sciences, Magadan, Russia; Erasmus University Medical Center, Netherlands

## Abstract

Following the dispersal out of Africa, where hominins evolved in warm environments for millions of years, our species has colonised different climate zones of the world, including high latitudes and cold environments. The extent to which human habitation in (sub-)Arctic regions has been enabled by cultural buffering, short-term acclimatization and genetic adaptations is not clearly understood. Present day indigenous populations of Siberia show a number of phenotypic features, such as increased basal metabolic rate, low serum lipid levels and increased blood pressure that have been attributed to adaptation to the extreme cold climate. In this study we introduce a dataset of 200 individuals from ten indigenous Siberian populations that were genotyped for 730,525 SNPs across the genome to identify genes and non-coding regions that have undergone unusually rapid allele frequency and long-range haplotype homozygosity change in the recent past. At least three distinct population clusters could be identified among the Siberians, each of which showed a number of unique signals of selection. A region on chromosome 11 (chr11:66–69 Mb) contained the largest amount of clustering of significant signals and also the strongest signals in all the different selection tests performed. We present a list of candidate cold adaption genes that showed significant signals of positive selection with our strongest signals associated with genes involved in energy regulation and metabolism (*CPT1A, LRP5, THADA*) and vascular smooth muscle contraction (*PRKG1*). By employing a new method that paints phased chromosome chunks by their ancestry we distinguish local Siberian-specific long-range haplotype signals from those introduced by admixture.

## Introduction

Hominins evolved in Africa for millions of years and adapted to survive in low latitudes and warm environments. After the dispersal out of Africa, over the past tens of thousands of years, however, our species has colonized almost all inhabitable climate zones of the world, including high latitudes and extremely cold environments. Siberia is one such region inhabited by humans today, being home to the coldest inhabited place in the world with a recorded minimum temperature of −71.2°C in winter. In present-day Siberia the climate varies dramatically over its different regions with an annual average temperature of −5°C. Archaeological evidence suggests that modern humans reached Southern Siberia by 45–40KYA [1], [2] expanding north as far as the Yana River Valley beyond the Arctic circle by 30KYA [3], while the earliest modern human remains in Siberia come from the Mal'ta site in Irkutsk Oblast date to 24KYA [2], [4]. During the Last Glacial Maximum (LGM) (26.5–19KYA) [5], the coldest period during the existence of modern humans, parts of Siberia were inhabitable [6]. If Siberian populations survived in this extreme climate for many thousands of years, it is possible that they have accumulated genetic changes that are adaptive in cold climates.

Technological and cultural innovations have certainly facilitated territorial expansion of humans in Siberia. Besides these, a number of morphological and physiological adaptations have been proposed to have evolved in Siberian populations in response to their environment. Low serum lipid levels were observed in indigenous Siberian populations, which are thought to be a consequence of increased energy metabolism and their elevated basal metabolic rate, BMR [7]. Central and Southern Siberian populations, Evenks, Yakuts, and Buryats also exhibit high blood pressure, higher than observed in most other circumpolar groups, including the North American and Greenland Inuit [8], [9], [10]. Thus it is likely that variants that permitted the ancestors of Siberian populations to adapt to colder climates may also have an important influence on the health of the present-day Siberian and possibly Native American populations.

Over the past few years, genome-wide analyses based on the HapMap [11], the Human Genome Diversity Panel (HGDP-CEPH) [12], [13] and the 1000 Genomes Project [14] data have significantly improved our understanding of human genetic diversity worldwide. However, there are still some crucial gaps in certain geographic regions, particularly those concerning populations living in areas of extreme climatic conditions such as Siberia. Previous studies [15], [16] have used genetic data from different Siberian populations to study origins of first Americans (including Greenland). A recent study [17] performed genome-wide scans for selection on 61 worldwide populations (including three Siberian populations), identifying SNPs with the strongest correlations between allele frequencies and nine climate variables. The study showed that the most extreme signals in worldwide populations came from SNPs associated with pigmentation and autoimmune diseases and pathways related to UV radiation, infection and immunity, and cancer. Notably, this study also found enrichment of strong correlations with climate variables for genes involved in the differentiation of brown adipocytes emphasizing the role of metabolic adaptations in populations living in cold climates. The role of genes involved in energy metabolism in cold tolerance was also shown in a previous study [18].

In this study, we introduce genome-wide genotype data for 200 individuals from ten different indigenous Siberian populations. We set out to identify regions in the genome that have been targets of positive natural selection. Several physiological functions have been associated with cold exposure and adaptation. On mild cold exposure, the body will try to conserve its heat by energetically inexpensive means such as vasoconstriction, piloerection, and by changes in posture to decrease surface area [19]. By the narrowing of subcutaneous blood vessels, vasoconstriction reduces peripheral blood flow and thereby preserves the core body heat. To protect tissues from cold injury the body resorts to a different process, cold-induced vasodilation, which increases the flow of warm blood near the skin surface [20]. While vasoconstriction leads to increased blood pressure which in turn leads to an increased heart rate (tachycardia) [21], it has been shown that cold-exposure may also reduce heart rate (bradycardia) [20], [22]. The different cardiac pressures involved during cold exposure might contribute to the increased mortality from coronary heart disease during winter [21]. In colder temperatures, when the heat conservation mechanisms are not sufficient, the body resorts to more active mechanisms of heat production such as non-shivering thermogenesis which occurs primarily in the brown adipose tissue (BAT) [19]. Norepinephrine initiates triglyceride breakdown in the brown adipocytes, leading to the release of fatty acids which activate the UCP1 enzyme leading to increased heat production [23]. All of these different physiological responses highlight the complex mechanisms involved in acclimatization to cold.

## Materials and Methods

### Samples, Genotyping, Quality Control and Phasing

We sampled 200 individuals from ten indigenous Siberian populations that form part of the DNA collection at the Genetics Laboratory, at the Institute of Biological Problems of the North, Magadan, Russia. The samples comprised 24 Buryats from Buryat Republic, 24 Evenks from the Krasnoyarsk region, 22 Yakuts from Sakha (Yakutia) Republic, 24 Shors from the Kemerovo region, 24 South Altaians (12 Altaian-Kizhi from Altai Republic, and 12 Teleuts from the Kemerovo region), 25 Koryaks from Severo-Evensk District of the Magadan Region, 24 Evens from Severo-Evensk and Ola Districts of Magadan Region, 14 Chukchi from Anadyr, Chukotka Autonomous Okrug and 19 Eskimos from Novoe Chaplino, Chukotka Autonomous Okrug. We also genotyped 18 Vietnamese individuals sampled in Singapore as a Southeast Asian reference group for our study. The study was approved by the Ethics Committee of the Institute of Biological Problems of the North, Russian Academy of Sciences, Magadan, Russia (statement no. 001/011 from 21 January, 2011) and Cambridge Ethics Committee (HBREC.2011.01). All subjects provided written informed consent for the collection of samples and subsequent analysis.

Samples were genotyped using Illumina OmniExpress Bead Chips for 730,525 SNPs. The data genotyped in this study has been deposited to the NCBI GEO repository and is accessible with GEO accession number GSE55586. Data filtering, quality checks and merging with other available data were performed in PLINK 1.07 [24]. The dataset was filtered to include only SNPs from the autosomes that had a genotyping success rate greater than 98%; 726,090 SNPs met this requirement. We also used genotype data from European (CEU) and Han Chinese (CHB) individuals from HapMap phase 3 samples [14] and Vietnamese (Southeast Asian) genotyped in this study to provide context for the analyses on Siberian data. The combined dataset included 672,684 SNPs.

Following quality control we phased autosomal SNPs using SHAPEIT [25]. All individuals were phased together with all HapMap phase 3 samples [14]. We estimated pairwise IBD iteratively using PLINK (excluding fixed alleles) on each population and removed individuals that had an IBD >0.125 in each iteration. This process was repeated until no individuals had an IBD >0.125. In the downstream analyses we further removed additional related individuals detected by ChromoPainter/fineSTRUCTURE as described below. In haplotype homozygosity tests we grouped individuals from various Siberian populations on the basis of their clustering by ADMIXTURE and fineSTRUCTURE. Minimum group size was kept to 20 to retain sufficient statistical power in the haplotype homozygosity tests [26]. Pooling samples from this and previously published studies using different genotyping platforms for the purpose of haplotype homozygosity tests was considered as undesirable because of major loss of SNP density. In the case of Population Branch Statistic (PBS) tests, which can be performed on lower SNP densities we merged our data with the Siberian samples from Rasmussen et al. [15], yielding a dataset of 302,693 SNPs. Simulations have shown that the haplotype homozygosity selection tests require at least 20 markers per 200 kb window [26] to have good power for the respective signal detection, we thus chose to retain our 672,684 SNP dataset for these analyses.

### Population Structure Analyses

To investigate population structure within the Siberian populations we used three commonly used methods: admixture analysis, model-based clustering, and Principal Component Analysis (PCA). ADMIXTURE 1.21 [27] was used to perform a maximum likelihood estimation of individual ancestries in our dataset. Each ADMIXTURE analysis requires a hypothesized number of ancestral populations (*K*) and assigns individuals the ancestry proportions using an unsupervised clustering method. We used European and Han Chinese populations from HapMap Phase 3 [14] as well as our Vietnamese data as reference populations. We thinned the dataset using PLINK by removing SNPs that were in linkage disequilibrium, with a pairwise *r^2^* value greater than 0.1 within a 50-SNP sliding window which was advanced by 10 SNPs each time [27]. This yielded a data set of ca. 70,000 SNPs which was used as an input to perform ancestry component analysis. We ran ADMIXTURE on this dataset 100 times for each *K* at *K* = 2 to *K* = 10 and analysed the cross-validation errors and log-likelihood estimates for each value of *K* to estimate the optimum number of *K* clusters.

To complement ADMIXTURE results and perform clustering of individuals we employed ChromoPainter/fineSTRUCTURE [28], [29]. We applied the ChromoPainter linked model on haplotypes of our unrelated Siberian and reference populations. GRCh37 recombination rates used in this analysis were downloaded from the HapMap website (http://www.hapmap.org). We performed EM inference using 10 EM steps to estimate the effective population size from our data and then used this estimated parameter in ChromoPainter. To perform Markov Chain Monte Carlo (MCMC) analysis for fineSTRUCTURE we used 5,000,000 burn-in iterations and sample iterations with a thin interval of 5,000. Visualisation of the posterior distribution of clusters was then performed using the tree-building algorithm of fineSTRUCTURE. Finally, PCA was performed on the normalized version of the coancestry matrix output from ChromoPainter using the fineSTRUCTURE GUI, which is an LD corrected version of standard PCA.

The population structure analyses furthermore allowed us to filter out additional outliers that did not group with their respective groupings and inbred individuals (more subtly related than those found by the PLINK IBD analysis) for use in subsequent analyses. For the haplotype-based tests we grouped Siberian populations into three groups such that the groups contain a reasonably homogenous set of individuals as implied by the population stratification analyses. The clustering of individuals followed, with the exception of a few outliers, the self-reported population and the geographic area of sampling: Southern Siberian group was composed exclusively of Altai-Kizhi, Teleuts, Shors and Buryats; the Central Siberian group of Evenks, Yakuts and Evens and the Northeast Siberian group of Koryaks, Chukchi and Eskimos. Simulations have shown that iHS and XP-EHH tests maintain power with sample sizes of ca. 40 chromosomes or above [26]. To maintain the minimum of 40 chromosomes in our test groups we grouped individuals by their genetic similarity using fineSTRUCTURE. For PBS tests we combined our data with Siberian data from Rasmussen et al. [15] to increase sample sizes and performed our downstream analyses on each Siberian population separately.

### Region Paintings

From our population structure analyses results we selected groups of unadmixed individuals that showed maximum divergence from other groups and used these as a panel representing “ancestral populations” to paint the phased chromosomes of all Siberian individuals. Individuals that showed no admixture with other ancestries from the ADMIXTURE analysis (≥0.99 in the ancestry coefficient matrix *Q* for the respective estimated ancestry) and which grouped in the same cluster in fineSTRUCTURE analysis were used for this purpose. This resulted in the identification of four different ancestral populations; Europeans, Vietnamese, unadmixed Central Siberian individuals (S1) and unadmixed Northeastern Siberian individuals (S2). These ancestral populations were used as donors in ChromoPainter [29] to paint admixed Siberian individuals. The unadmixed Siberian individuals were painted by conditioning unadmixed individuals against each other and summing up the expected probabilities from individuals coming from the defined ancestral population. Painting plots were produced by assigning each SNP to an ancestor population X if the expected copying probability for X>0.7. Any SNPs with an expected probability of ≤0.7 were marked as undecided.

### Admixture dating

**Figure 1:**
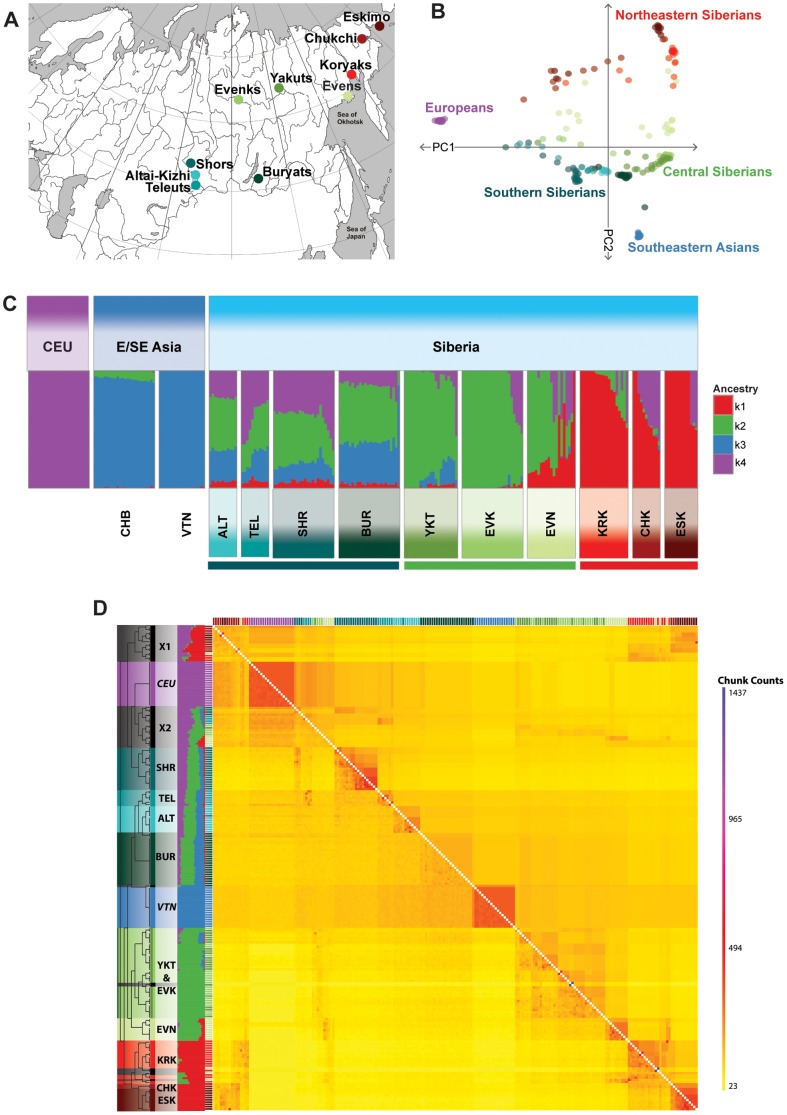
Population structure analyses in Siberian populations. (A) Averaged sampling locations of the Siberian populations genotyped in this study. (B) Principal component analysis of Siberian populations and reference populations from West Asia (European) and Southeast Asia (Vietnamese). Each dot in the plot represents an individual. The PC axes were rotated 180 degrees anti-clockwise to emphasize the similarity to the geographic map of Eurasia. (C) ADMIXTURE analysis at *K* = 4. (D) Coancestry heatmap for the Siberian individuals and reference populations (Europe, Vietnamese) output by ChromoPainter/fineSTRUCTURE. The heatmap shows the number of shared genetic chunks between the individuals. The raw data is shown on the bottom left and the aggregated data is shown on the upper right of the heatmap. Adjacent to the heatmap is also the ADMIXTURE plot of the respective individuals. To the left is the maximum a posteriori (MAP) tree generated by fineSTRUCTURE which shows the groupings of the different populations. The following abbreviations are used in the Figure: ALT, Altai-Kizhi; BUR, Buryats; CEU, European; CHB, Han Chinese; CHK, Chukchi; E/SE Asia, East/Southeast Asia; ESK, Eskimo; EVN, Evens; EVK, Evenks; KRK, Koryaks; SHR, Shors; TEL, Teleuts; VTN, Vietnamese; X1, Northeastern Siberian admixed individuals; X2, Southern and Central Siberian admixed individuals; YKT, Yakuts. Reference populations are labelled in italics. Outliers removed in downstream analysis are blacked out in the tree.

In order to evaluate the admixture scenarios suggested by the ADMIXTURE plots, we tested all possible sets of recipient and source populations with the three population test (*f3*) [30], [31]. The Siberian samples were grouped according to their self-reported ethnicity and the grouping suggested by fineSTRUCTURE analysis [29]. We also included the admixed Siberian clusters X1 and X2 (Figure 1D) in the analysis. The population trios that yielded a Z-score smaller than −2 in the *f3* test were considered as significantly admixed, and a subset of them were subsequently analysed with ALDER [32] to determine the time elapsed since each putative admixture event. The admixture events were dated with ALDER using default parameters with Mindis  = 0.005 using as source populations a combination of the following: CEU, CHB (HapMap), unadmixed Central Siberian individuals (S1), unadmixed Northeastern Siberian individuals (S2 - as described in the previous section) and Vietnamese (our samples). From this subset we report the admixture dates that were successful.

### Scans of positive selection

We used the Integrated Haplotype Score (iHS) [33] and Cross Population Extended Haplotype Homozygosity (XP-EHH) [34] tests for capturing haplotype homozygosity based signals of positive selection. Previous power analyses have suggested that iHS has maximum power to detect selective sweeps that have reached moderate frequency (ca. 50%–80%); while XP-EHH has good power to detect selective sweeps at high (80–100%) frequency [26], [33], [34], thus making the two tests complementary.

Both the iHS and XP-EHH statistics were calculated as in Pickrell et al. [26] using tools available at http://hgdp.uchicago.edu. HapMap GRCh37 genetic map was used to calculate genetic distances between markers. Ancestral states were retrieved from the Ensembl Variation database rel. 68. iHS and XP-EHH were computed for autosomal SNPs. SNPs with inferred ancestral states and a minor allele frequency of at least 5% were used for iHS. This setup yielded ca. 11,000 windows for iHS and ca. 13,000 windows for XP-EHH, for each Siberian population group. Vietnamese were used as an outgroup population for the haplotype homozygozity tests as no degree of admixture with a Siberian specific ancestral component was observed in the Vietnamese samples (Figure 1). For iHS, the top 1% windows present in each Siberian group were identified as our candidate selected regions excluding windows present in the top 5% iHS windows of our outgroup population (Vietnamese). XP-EHH was calculated using Vietnamese as a reference population for all comparisons. These comparisons are likely to reveal selective sweeps that happened recently and are not widespread through East Asia. The top 1% of XP-EHH windows present in each Siberian group were identified as our candidate selected regions from this test.

We also computed a SNP-based test; the Population Branch Statistic (PBS), which represents the amount of allele frequency change at a given locus in the history of the test population since its divergence from other populations [35]. PBS requires three populations; a test population and two outgroup populations that have a specified evolutionary relationship. We excluded all populations with less than 10 samples from the analysis. Pairwise-*F_ST_* was estimated by a “weighted” analysis of variance [36], [37] using GENEPOP'007 [38]. PBS scores were estimated from the pair-wise *F_ST_* values as in Yi et al. [35]. We divided the genome into 100 kb windows and used the maximum PBS score in the window as our test statistic, adapting the approach from Pickrell et al. [26]. This setup yielded ca. 26,300 windows. The top 1% windows with the highest PBS test statistic were identified as our candidate regions under selection.

Our top windows from the selection tests were mapped to genes using the Ensembl Genes Human database rel. 68. Manhattan, ADMIXTURE, PCA and regional paintings plots were drawn using the ggplot2 [39] and Bioconductor [40] packages in R [41].

### Gene Enrichment

We performed window enrichment analysis looking for increased representation in the top 1% iHS and XP-EHH windows of biological functions that could potentially be involved in cold adaptation. We used Gene Ontologies (GO-terms) [42] to denote biological functions and applied a modified version of the DAVID algorithm [43], [44] that takes into consideration window counts instead of gene counts and thus corrects for positional clustering [45], such that no GO term is associated with any window more than once. We used the EASE score [45] as a conservative adjustment to the Fisher's exact test which favours biological functions that involve more windows. We have considered correcting the EASE score for multiple testing by applying Benjamini FDR correction [46]. However, since other evidence suggests that it may not improve specificity [44] and the sensitivity may actually be negatively affected due to its conservative nature [47], we ultimately used the EASE score uncorrected for multiple testing to denote a category as significantly enriched. We therefore classified a biological process as significantly enriched if the EASE score was ≤0.01.

Since PBS is an allele frequency based test, we identified the protein-coding genes containing SNPs exhibiting the highest PBS scores from our top 1% PBS window data and used these genes as input to DAVID [43], [44] to uncover the biological processes enriched in our PBS results. We screened the GO biological processes and analysed the enriched clusters from DAVID. As with iHS and XP-EHH gene enrichment analysis, we used significant EASE scores (p-values ≤0.01) to uncover significant biological processes from our PBS scores.

### Definition of Gene Lists

We generated a cold adaptation-related seed gene list by considering genes associated with phenotypic features that have been attributed to cold adaptation. These include basal metabolic rate, non-shivering thermogenesis, response to temperature, smooth muscle contraction, blood pressure and energy metabolism using relevant Gene Ontology terms, pathways and experimental evidence while defining the lists.

### Comparison with other studies

We used published results from a study that examined selection in response to 9 climatic variables in a panel of 61 worldwide populations, including three from Siberia (Maritime Chukchi, Naukan Yu'pik (Eskimos) and Yakut) [17]. These data were obtained from http://genapps2.uchicago.edu:8081/dbcline/climate.tar.gz. Since we are interested in cold adaption selection signals we used the minimum winter temperature variable for comparison. We extracted the strongest signals with transformed ranked signals <5×10^−4^ and mapped these signals to our top 1% iHS, XP-EHH and PBS selection regions for all Siberian populations. Loci were mapped to genes using the Ensembl Genes Human dataset rel. 68.

## Results

### Population structure analyses and admixture dating

We genotyped 200 Siberian individuals from ten indigenous Siberian populations across the Siberian landscape (Figure 1A) and called successfully 726,090 autosomal SNPs. As some of the selection tests we use are based on haplotype homozygosity patterns we searched for closely related individuals (IBD >0.125) and removed 23 individuals from downstream analyses. (Tables S1 and S2 in File S2). To control for the effect of population structure and recent admixture on the selection tests we performed further analyses and filtered out outliers and individuals with high proportion of recent European admixture.

Firstly, we used PCA to reveal levels of genetic differentiation and population structure in the Siberian populations in the broader Eurasian context (Figure 1B, Figure S1B in File S1). The first component separates populations on the east/west axis. The two reference populations form tight clusters while a number of Siberian samples are diffused along intermediate coordinates. PC2 reflects north and south differences and contrasts most clearly Northeast Siberians with the Southeast Asian reference population (Vietnamese). Central and Southern Siberian populations cluster between them. Some individuals from various Siberian populations are widely dispersed away from the population average. One likely cause for such clinal patterns is recent European admixture. Since recent admixture can create admixture-LD [48], which can significantly confound haplotype homozygosity-based scans of selection, we examined the Siberian genotype data further with ADMIXTURE and ChromoPainter/fineSTRUCTURE to identify such individuals and exclude them from our downstream analyses.

To estimate the optimum number of clusters *K* used in ADMIXTURE analysis, we ran ADMIXTURE 100 times using *K* values from 2 to 10 in each iteration. The minimum cross-validation error in the ADMIXTURE analysis was observed at *K* = 4. The difference between maximum and minimum log-likelihood scores over all 100 iterations was 0 at *K* = 4 suggesting that the log-likelihood score had reached its global maximum (Figure S2 in File S1). Three different ancestry profiles could be distinguished among native Siberian populations (Figure 1C, Figure S1C in File S1): one with predominantly k1 (Chukchi, Eskimo, Koryaks), one with k2 ancestry (Yakuts, Evens, Evenks), and one with a mixture of all four components (Altaian-Kizhi, Teleuts, Shors and Buryats). These results were largely consistent with the ancestry profiles at *K* = 6, *K* = 5 and *K* = 3 (Figure S3 in File S1). The population structure at *K* = 3 clustered populations between European, East/Southeast Asian and general Siberian components. Consistent with the PCA results the West Eurasian component was the most variable within populations suggesting recent admixture. This was confirmed by ALDER analysis, that dated European admixture in the Central and Northeastern Siberian populations from ∼6 to ∼3 generations ago respectively (Figure S4 in File S1, Table S3 in File S2). At *K*≥5 we noticed a separate component emerging that separates the Shors from their neighbouring populations of Altai-Kizhi, Teleut and Buryat. In ALDER analysis Shors exhibited more ancient admixture dating than the neighbouring Siberian populations when using Europeans and ancestral Siberian groups (S1 and S2) and other Asian populations (Han Chinese, Vietnamese) as ancestor populations. Furthermore, ADMIXTURE analysis revealed that Evens contain both k1 and k2 components consistent with the geographic area of their sampling since they are located in between the populations that are largely composed from the k1 and k2 components.

We also performed analyses with ChromoPainter/fineSTRUCTURE [28], [29] which allowed us to cluster individuals based on their genetic similarity. Unlike ADMIXTURE, ChromoPainter takes into consideration LD patterns in the genome thus extracting more information from the data. The coancestry heatmaps (Figure 1D, Figure S1D in File S1) show the amount of shared genetic chunks between the different Siberian individuals and reference populations. Utilizing the fineSTRUCTURE results we identified Siberian individuals that did not group with their respective counterparts or showed similar patterns and excluded them from our downstream analyses. These outliers were further confirmed when mapping ADMIXTURE results to the coancestry heatmap (Figure 1D), which also showed different patterns from their respective counterparts. Moreover, through ChromoPainter we identified as outliers additional inbred individuals that had not been detected by IBD filtering (Tables S1 and S2 in File S2). These were also excluded from our downstream analyses, leaving homogenous groups of unrelated individuals, consistent with their geographical locations to be used in our tests of selection.

### Selection Tests

Long term habitation in an extremely cold environment can be expected to result in biological adaptations that may affect the distribution of allele frequencies and homozygosity patterns. To examine this hypothesis we employed haplotype-based tests - iHS [33] and XP-EHH [34] - and a SNP allele frequency spectrum test (PBS) [35] on our Siberian data. The genome-wide top 1% iHS, XP-EHH and PBS results are reported in Figures S5–S6 in File S1 and Tables S4–S7 in File S2. We first calculated the fraction of overlapping iHS, XP-EHH and PBS windows (Figure S7 in File S1). For a window to be considered overlapping between two populations, we used the criteria set by Pickrell et al. [26] that required the window to be present in the top 1% of one population and the top 5% of the other. The results in Figure S4 in File S1 are consistent with the population structure results (Figure 1, Figure S1 in File S1) in that populations geographically closer to each other tend to share more windows with each other rather than with the more distant ones. In haplotype homozygosity tests southern and central Siberian populations shared 40–62% of the selection signals, while the Northeast Siberians shared only 18–26% of their iHS and 27–34% XP-EHH signals with the other two groups. In PBS tests the highest sharing was observed between Evenks and Yakuts (68%) while the sharing among other populations was relatively low (12–51%). Indeed this was also reflected in the results of ChromoPainter and fineSTRUCTURE where Evenks and Yakuts clustered together (Figure 1D, Figure S1D in File S1). Overall, the level of selection signal sharing among Siberian populations was found to be lower than within other continental regions [26], [49].

To gain insights into biological processes targeted by selection in Siberian populations, we performed GO term enrichment analysis of the top 1% results of each selection test. These tests revealed 88 GO terms (EASE score ≤0.01), none of which was significant after FDR correction (Tables S8–S9 in File S2). The enriched categories were mostly generic terms, including response to stress, metabolic processes, growth, development and immune function. However, since positive selection does not necessary lead to gene set enrichment, we examined the top ten regions from each selection test. To identify the candidate genes involved in cold adaptation, we searched for the presence of genes present in the pre-defined seed gene list of cold adaptation (Table S10 in File S2) among the upper tails of the selection tests (Tables S4–S6 in File S2). Amongst our strongest signals (top 10 ranking windows), we found seven genes (*THADA, ITPR3, GNGT1, PRKG1, RELN, CPT1A* and *LRP5*) that were also present in the pre-defined cold adaptation seed gene list that was detected by at least one of the tests (Table S11 and S12 in File S2). The latter four of these genes were also significant (top 1%) by one additional test (Table S7 in File S2). No enrichment (P>0.2; Fisher's exact one-sided test) of the pre-defined cold adaptation genes was found in the top 10 windows of the different selection tests.

**Figure 2:**
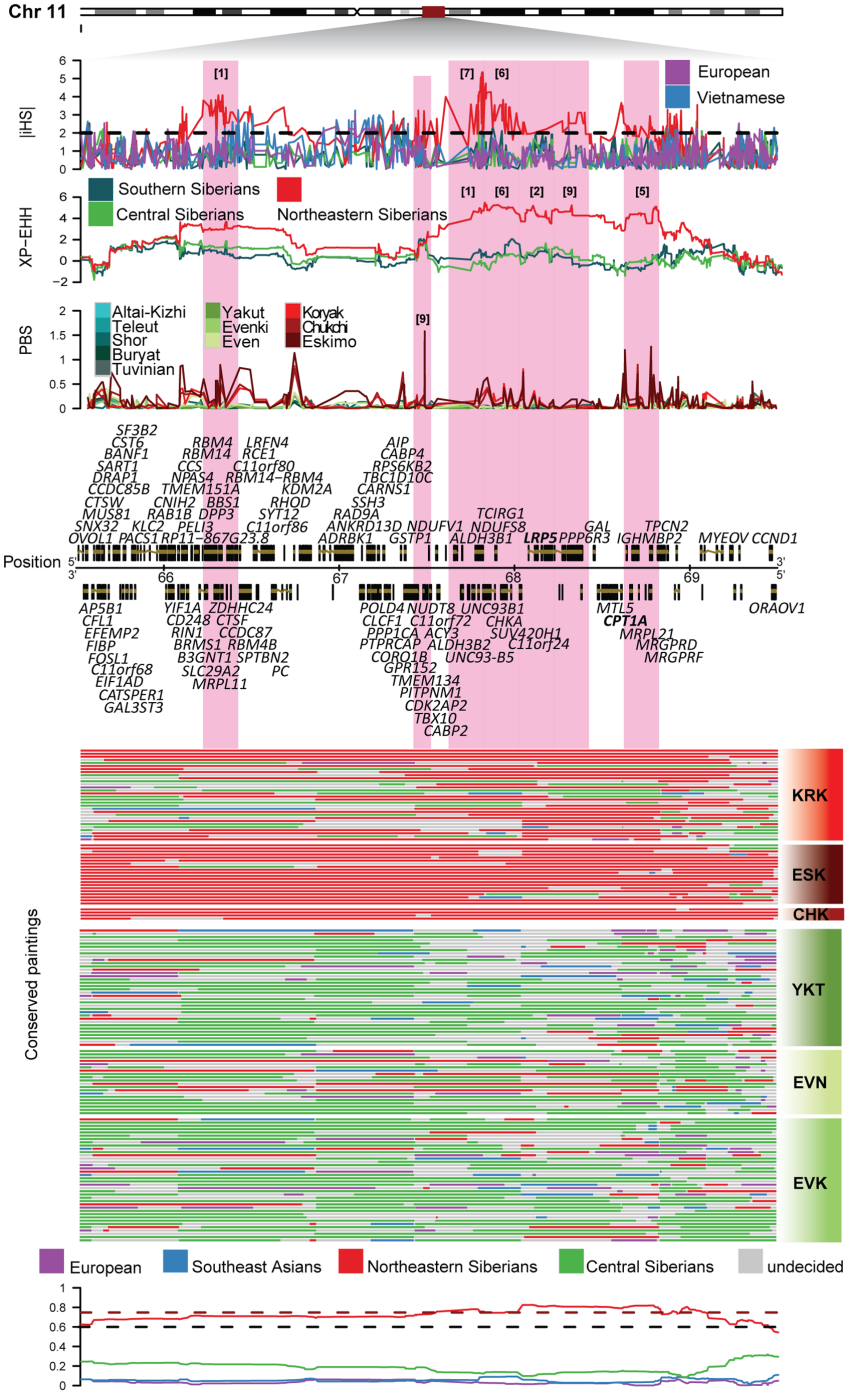
Zooming on the region on chromosome 11 that showed the strongest signals in the Northeastern Siberian populations. The |iHS|, XP-EHH and PBS scores that showed strong signals (amongst top 10 ranking windows) in the Northeastern Siberian populations are shown in the upper panels for the different Siberian populations. The pink highlights mark the windows present in the top 10 ranking windows in the Northeastern Siberian populations. The rankings are marked on the respective windows in the respective test panels. Protein-coding genes present in the 4 Mb region are shown under the test plots with the genes present in our predefined cold adaptation list marked in bold font (*CPT1A* and *LRP5*). The position on the chromosome is given in Mb. The paintings of the phased chromosomes for the region in the Northeastern and Central Siberian individuals are shown underneath the *Position* legend. The aggregated ancestral probabilities from ChromoPainter for the Northeastern Siberian individuals are displayed below the paintings. The dotted red line shows the threshold for the upper 5% tail (0.75) and the black dotted line shows the mean of the genome-wide probabilities distribution of the Northeastern Siberian ancestor (red).

**Figure 3:**
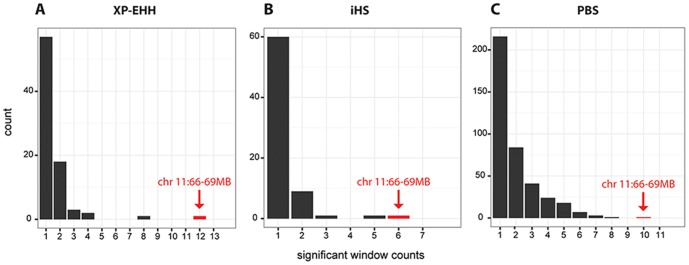
Genome-wide distribution of significant selection windows. The distribution of the significant windows of the three different selection tests results (A) XP-EHH, (B) iHS and (C) PBS for the Northeastern Siberian populations. The x-axis show the count of significant windows (Tables S4–S6 in File S2) per 3 Mb region and the y-axis show the amount of 3 Mb regions that had the respective count displayed on the x-axis. In all the different selection tests, the region on chromosome 11:66–69 Mb is the region with the most significant selection windows (highlighted in red).

The strongest selection signals over all tests map closely in a 3 Mb region (chr11:66–69 Mb) of chromosome 11 (Figure 2) which showed, overall, the highest concentration of significant hits by different selection tests (Figures 2–3) in the Northeastern Siberian populations. This region contains two cold adaptation candidate genes; *CPT1A* and *LRP5*. We mapped the significant windows over all the genome to 3 Mb regions for all the three different selection tests and the largest amount of significant window counts was observed at the same region; chr11:66–69 Mb (Figure 3). We used ChromoPainter to determine whether this chromosome 11 region with unusually high haplotype homozygosity shows predominantly local or admixed ancestry in Northeastern Siberians and whether the two highlighted genes derive from shared or different ancestry chunks. Painting of the phased chromosomes by their ancestry in the 3 Mb region surrounding the *CPT1A* and *LRP5* genes revealed that most chromosomes sampled from Chukchi and Eskimo populations and a substantial proportion of Koryak chromosomes share the most likely ancestry in their local Northeastern Siberian gene pool in the region (P-value <0.05). Since the region contains several other protein coding genes (which were not in our cold adaptation candidate gene list) which lie in the same block of high homozygosity and the same ancestry, it is not possible from the given data to distinguish which gene, in particular, drives this signal.

The results of a previous genome-wide scan involving 61 worldwide populations and their correlations between allele frequencies and climatic variables [17] have provided us with the opportunity to compare these previously reported signals with our top regions of positive selection. From all the 61 worldwide populations analysed, Yakut and Maritime Chukchi (both populations present in our dataset) experience the lowest minimum temperatures. We mapped candidate signals that were associated by Hancock et al. [17] with the minimum winter temperature to our top 1% iHS, XP-EHH and PBS regions of selection of our Siberian populations. Genes present in the overlap provide a candidate list of cold adaptation genes that are also present in other populations. There was an overlap of 4% in iHS, 6% in XP-EHH and 13% in PBS tests (Table S13 in File S2). Amongst the genes present in this list that also exhibited high scoring signals in our analysis (6^th^ ranking XP-EHH signal) was *POLD3* that encodes a subunit of DNA polymerase delta which is involved in DNA damage response produced by UV irradiation [50]. Windows containing *POLD3* gene show high signals in all selection tests with the highest signals coming from the southern Siberian populations (Figure S8 in File S1).

## Discussion

In this study we have performed systematic genome-wide scans of extended haplotype homozygosity, and allele frequency differentiation among Siberian populations. In contrast to previous studies that have found high levels of selection signal sharing across wide geographical areas in Eurasia [26] the majority of selection candidate regions detected in this study turned out to be specific to regional subgroups of Siberian populations, or even specific to individual populations (Figure S7 in File S1). The level of iHS signal sharing among Siberian groups (18–47%; Figure S7 in File S1) was, in fact, comparable to that observed among distinct geographic regions, e.g. between Europe and South Asia (37–55%), and between Europe and East Asia (21–22%) [49]. These results are meaningful in the light of the low effective population sizes and vast geographic areas inhabited by the Siberian populations.

Consistent with the results of previous studies [15], [51] our analyses revealed that the genetic landscape of Siberian populations is characterized by two main components (k1 and k2, Figure 1C). It is not obvious from these analyses whether k1 and k2 represent two separate population dispersals, or result from long term genetic isolation and low effective population size of the Siberian populations. One of these components, k1, is shared by Northeast Siberian and North American and Greenland Inuit populations [15]. An example of a more complex pattern of population structure was found in the Southern Siberian group that showed presence of West and East Eurasian ancestry components. A gradual cline of European admixture dating can be noticed from western to eastern Siberia (Figure S4 in File S1) with the most recent European admixture estimated in the Northeastern Siberian populations (∼3 generations ago) which is consistent with the industrial development of these territories. Moreover, Buryats showed evidence of East Asian admixture (∼33–37 generations ago) which dates back to the Mongolian conquest of Southern Siberia era. In the selection tests we have observed that signal sharing among populations follows the patterns detected in the analyses of population structure and therefore it is unsurprising that only a minority of high ranking selection signals turned out to have pan-Siberian distribution.

Haplotype homozygosity patterns in the genome are affected by demographic history and selection for different environmental factors including diet, disease and climate. Being one of the oldest populations inhabiting extreme cold environments and ancestors to other Arctic populations, in this study, we highlight the signals that we hypothesise to be a consequence of cold adaptation in indigenous Siberian populations. We note however, that other factors, such as their particular diet, could also have shaped the variation we detected in their genomes. Cold adaptation and acclimatization studies suggest that there is more than one mechanism involved in the biological response to cold stress. Consistent with the expectations from the biological complexity of cold adaptation several different processes rather than one particular term or pathway were highlighted by our selection tests. In fact, none of the enrichment tests provided significant results after correcting for multiple testing. This lack of significant enrichment could be explained by the fact that the selection tests we used are designed to detect hard sweeps at individual loci that may be generally rare while soft sweeps affecting large number of functionally related genes would require different approaches of detection [52]. Soft sweep detection on the basis of small scale allele frequency differences at multiple loci requires high resolution SNP-level functional annotation for a phenotype of interest, e.g. as available for more than 180 height associated loci in Europe [53]. As no such SNP lists exist yet according to our knowledge for cold adaptation phenotypes, relevant soft sweep tests on Siberian populations cannot be performed yet. However, because systematic hard sweep scans on Siberian populations have not been performed, we analysed the Siberian genome-wide data with three different selection tests that complement each other and reveal the different signals under selection in the different Siberian populations by focusing on the strongest regions (present in top ten windows of a selection test and also significant in other selection test) that contain genes present in our pre-defined candidate list of cold adaptation genes.

The strongest signals mapped to chromosome 11:66–69 Mb region that contains a cluster of seven top 10 ranking windows over all three tests which also included two cold adaptation candidate genes, *CPT1A* and *LRP5* (Figure 2). Moreover, the genome-wide distribution of significant window counts (Figure 3) showed the largest number of counts in the same region of chromosome 11:66–69 Mb in all the three different selection tests. This persistent clustering of significant selection signals in the region containing *CPT1A* and *LRP5* that also contains the topmost signals, highlights the importance of the region. Evidence of selection in this 3 Mb region was uniquely strong in Chukchi and Eskimo populations who reside in the Northeastern coast of Siberia. *CPT1A* encodes a liver isoform of carnitine palmitoyltransferase IA that is involved in the metabolism of long-chain fatty acids. *LRP5* (low density lipoprotein receptor-related protein 5) has similarly the highest expression in the liver and plays a role in bone growth [54], cholesterol metabolism, systolic blood pressure and adrenarche [55]. Considering the role of the two highlighted genes in lipid metabolism, it is possible that an efficient energy regulation process could have evolved in these populations to help them cope with the extreme cold climate. It is also possible that the selection signal is somehow associated with the high fat diet of Northeastern Siberian populations who have to cope with ketosis [56], [57]. The high fat diet is in itself a direct consequence of cold adaptation since crop growing is not sustainable in the arctic climate, thus these populations had to use other sources for subsistence; mainly from organisms that live in cold climates such as reindeer, seal, walrus and whale [58] that are in close proximity to these populations' habitats. Other previous genome-wide selection scans have already highlighted a number of diet related genes as targets of hard sweep, e.g. *LCT* [59]. Even though their diets are rich in animal food and fat [57] indigenous Siberians have relatively low serum cholesterol and lipid levels[7], suggesting that diet related and metabolic adaptive processes may have played an important role in the evolution of Siberian populations. These adaptive processes would primarily be expected to involve fatty acid metabolism as continuous cold exposure is known to determine the mobilization and metabolism of fat as energy and heat source [20]. However, as both *CPT1A* and *LRP5* genes mapped to the same high haplotype homozygosity block along with several other genes further sequencing data from this genomic region will be required to determine which gene carries functionally significant variants that may be driving the selection signal.

The fact that indigenous Siberian groups have significantly elevated basal metabolic rate may also play a role in the maintenance of stable lipid levels in the serum. It has been observed that mice consuming ketogenic diets, characterised by a high content of fat and low carbohydrates, had increased metabolic rates while their serum lipids did not increase [60]. These mice also exhibited an overexpression of *UCP1* and *UCP2*, suggesting activation of non-shivering thermogenesis which uncouples the mitochondrial respiration by impairing ATP production and dissipating energy as heat. Previously, Leonard et al. [7] have hypothesized that the elevated basal metabolic rates in Siberian populations are due to genetic adaptations in their thyroid hormone signalling pathway. Recent studies suggest that thyroid hormone mediated thermogenesis emanates from the brown adipose tissue [19], [61] providing a link between BMR and non-shivering thermogenesis. It has been shown that *CPT1A* is regulated by thyroid hormone and insulin [62] highlighting the imperative role the gene has in energy regulation. Another gene associated with thyroid function that was highlighted in our selection scans as top 10 ranking PBS signal in two south Siberian populations is *THADA* (Tables S6 and S12 in File S2) which is one of the few genes that has been confirmed to be significantly associated with Type 2 Diabetes in multiple studies; in European, Asian, and Native American cohorts [63], [64]. Notably, *THADA* was also highlighted as a gene with unusually low diversity in Neanderthals when compared to humans [65], suggesting that variation in this gene may have affected aspects of energy metabolism in early modern humans.

Smooth muscle contraction which includes vasoconstriction and vasodilation is another process implicated in cold acclimatization. One of the genes involved in these processes that showed evidence of strong signals of selection is *PRKG1*. Though in PBS some significant signals were also found in the central Siberian populations, the strongest signals considering all tests are present in the Northeastern Siberian populations (Figure S9 in File S1). The PRKG proteins play a central role in regulating cardiovascular and neuronal functions in addition to relaxing smooth muscle tone, preventing platelet aggregation, and modulating cell growth [66], [67]. They also act as a mediator of nitric oxide/cGMP signalling pathway. While no data on the heart rate of the Siberian populations is available, it is known that cold exposure increases cardiac pressure [20], [21], [22], thus efficient cardiovascular regulation in cold climates could be seen as a possible adaptive mechanism in the indigenous Siberian populations.

Mechanisms allowing adaptation to the cold environment are expected to be complex and probably similar to convergent processes of high altitude adaptation [68] and therefore different cold-adapted populations may have undergone selection processes targeting different physiological functions [19], [21]. Our results show that the signals of genetic selection detected in Siberian populations are not uniformly spread among populations and different Siberian groups show different selection signals. Factors affecting the different populations such as their particular ways of subsistence and the varied climates they are exposed to could be the main cause of the genetic variation observed. Also, demographic histories of Siberian populations, which to date are still not well understood, might play an important element in explaining the observed genetic variation. Many high ranking genome regions in our selection tests have not yet been associated with any specific phenotype and thus require further analyses at the sequence level to determine their role, in biological processes involved in the adaptation of native Siberian over thousands of years to their extreme environment.

## Supporting Information

File S1 **This file contains Figure S1 through Figure S9.** Figure S1, Population structure analyses in Siberian populations when merged with Rasmussen et al.'s study. Figure S2, ADMIXTURE CV-error and log likelihood scores. Figure S3, ADMIXTURE analysis for K = 3 to 6 for the Siberian populations and representatives of East and West Eurasia. Figure S4, Admixture dating of Siberian populations and groups of admixed individuals. Figure S5, Genome-wide plot of empirical -log10 P-values of window haplotype-homozygozity scores for all Siberian groupings. Figure S6, Genome-wide plots of maximum PBS scores in windows in the Siberian populations. Figure S7, Window sharing between Siberian groups in regions. Figure S8, Zooming on the 2 Mb region around the POLD3 gene. Figure S9, Zooming on the 2 Mb region around the PRKG1 gene.
(PDF)

File S2 **This file contains Table S1 through Table S13.** Table S1, Sample sizes of Siberian populations that were genotyped in this study. Table S2, Sample sizes of Siberian populations used in PBS tests. Table S3, Admixture dating for Siberian populations/groups. Table S4, Empirical ranks of the top 1% iHS windows in three Siberian population groups. Table S5, Empirical ranks of the top 1% XP-EHH windows in three Siberian population groups. Table S6, Empirical ranks of the top 1% PBS windows in Siberian populations. Table S7, Top 10 windows present in more than one selection test. Table S8, Window enrichment analysis of the top 1% ranking genes by haplotype homozygosity tests in three Siberian population groups. Table S9, Gene enrichment analysis of the genes with maximum PBS present in the top 1% windows in all Siberian populations. Table S10, Definition of gene lists hypothesized to be involved in cold adaptation. Table S11, Cold-adaptation genes among the top 1% ranking windows by haplotype homozygozity tests. Table S12, Cold-adaptation genes found in top 1% ranking PBS windows. Table S13, Significant windows in the iHS, XP-EHH and PBS tests that intersect with the significant SNPs for minimum winter temperature variable in Hancock et al. 2011 study.
(XLSX)
