# Supplementary material for: Genome-Wide Analysis of Cold Adaptation in Indigenous Siberian Populations
Source: PLoS One. 2014 May 21;9(5):e98076. doi: 10.1371/journal.pone.0098076 (PMC4029955; doi:10.1371/journal.pone.0098076)
Supplement: File S1 — This file contains Figure S1 through Figure S9. Figure S1, Population structure analyses in Siberian populations when merged with Rasmussen et al.'s study. Figure S2, ADMIXTURE CV-error and log likelihood scores. Figure S3, ADMIXTURE analysis for K = 3 to 6 for the Siberian populations and representatives of East and West Eurasia. Figure S4, Admixture dating of Siberian populations and groups of admixed individuals. Figure S5, Genome-wide plot of empirical -log10 P-values of window haplotype-homozygozity scores for all Siberian groupings. Figure S6, Genome-wide plots of maximum PBS scores in windows in the Siberian populations. Figure S7, Window sharing between Siberian groups in regions. Figure S8, Zooming on the 2 Mb region around the POLD3 gene. Figure S9, Zooming on the 2 Mb region around the PRKG1 gene. (PDF) [file pone.0098076.s001.pdf]

Supplementary Figures for

Genome-wide analysis of cold adaptation in indigenous Siberian populations

Alexia Cardona[1]*, Luca Pagani[1,2], Tiago Antao[3], Daniel J. Lawson[4], Christina A. Eichstaedt[1], Bryndis Yngvadottir[1], Ma Than Than Shwe[5], Joseph Wee[5], Irene Gallego Romero[6], Srilakshmi Raj[7], Mait Metspalu[8,9], Richard Villems[8,9], Eske Willerslev[10], Chris Tyler-Smith[2], Boris A. Malyarchuk[11], Miroslava V. Derenko[11], Toomas Kivisild[1,9]

Affiliations:

[1] Department of Archaeology and Anthropology, University of Cambridge, Cambridge, United Kingdom

[2] Wellcome Trust Sanger Institute, Hinxton, United Kingdom

[3] The Wellcome Trust Centre for Human Genetics, University of Oxford, Oxford, United Kingdom

[4] Department of Mathematics, University of Bristol, Bristol, United Kingdom

[5] National Cancer Centre Singapore, Singapore

[6] Department of Human Genetics, University of Chicago, Chicago, United States of America

[7] Department of Molecular Biology and Genetics, Cornell University, Ithaca, United States of America

[8] Department of Evolutionary Biology, Institute of Molecular and Cell Biology, University of Tartu, Tartu, Estonia

[9] Estonian Biocentre, Tartu, Estonia

[10] Centre for GeoGenetics, Natural History Museum of Denmark, University of Copenhagen, Copenhagen, Denmark

[11] Institute of Biological Problems of the North, Russian Academy of Sciences, Magadan, Russia

*Correspondence to: ac812@cam.ac.uk

This PDF file includes:

Figures S1 to S9

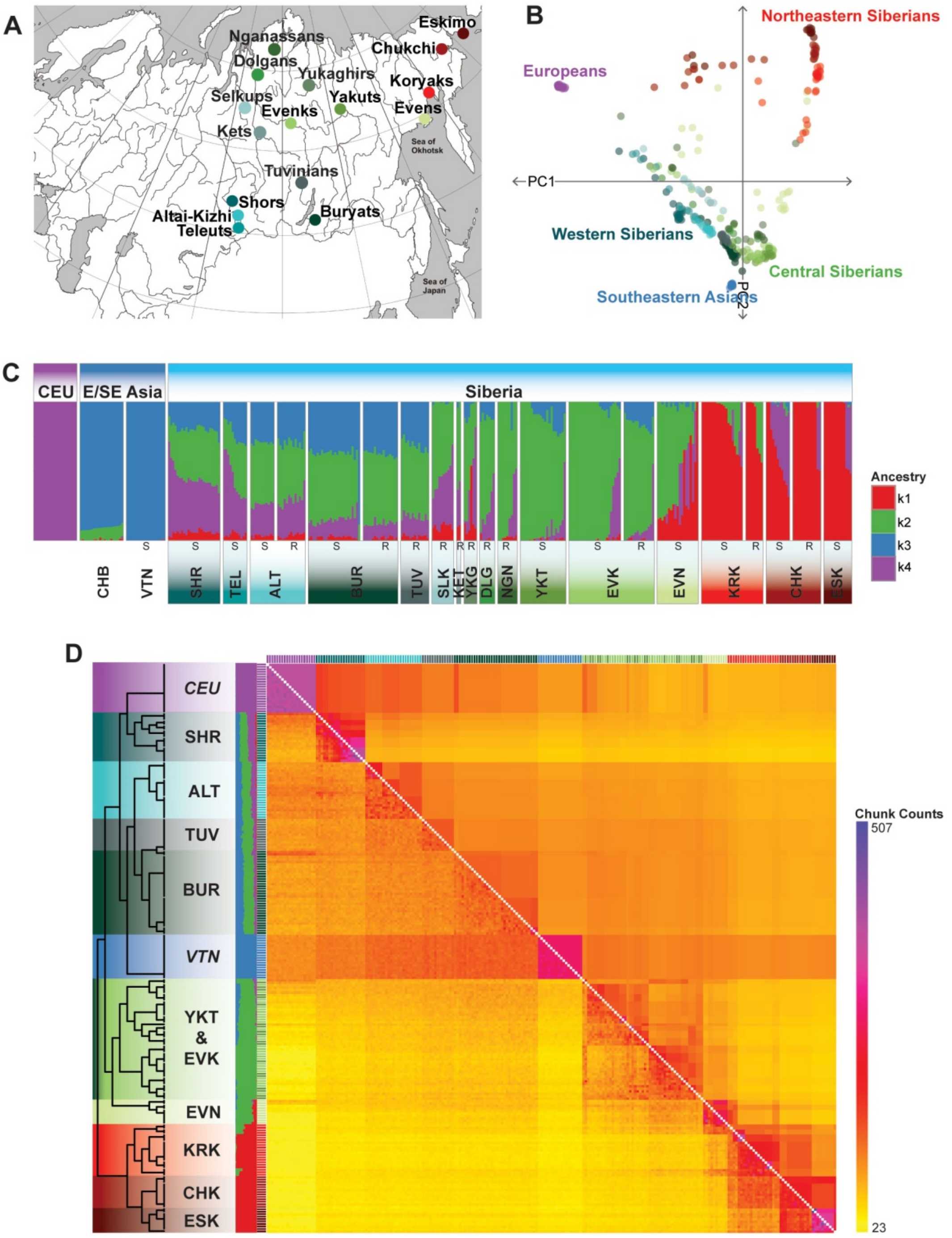

Figure S1. Population structure analyses in Siberian populations when merged with Rasmussen et al.'s study.
(A) Averaged sampling locations for the Siberian populations genotyped in this study and locations of populations from Rasmussen et al.'s study. (B) Principal component analysis of Siberian populations and reference populations from West Asia (European) and Southeast Asia (Vietnamese). Each dot in the plot represents an individual. The PC axes were rotated 180 degrees anti-clockwise to emphasize the similarity to the geographic map of Eurasia. (C) ADMIXTURE analysis at K=4. Each vertical line represents an individual. Individuals marked with *S* refer to individuals from our study; individuals marked with *R* refer to individuals from Rasmussen et al.'s study. (D) Coancestry heatmap for the Siberian individuals and reference populations (Europe, Vietnamese) used in our downstream analysis output by ChromoPainter/fineSTRUCTURE. The heatmap shows the number of shared genetic chunks between the individuals. The raw data is shown on the bottom left and the aggregated data is shown on the upper right of the heatmap. Adjacent to the heatmap is also the ADMIXTURE plot of the respective individuals. To the left is the maximum a posteriori (MAP) tree generated by fineSTRUCTURE which shows the groupings of the different populations. The following abbreviations are used in the Figure: ALT, Altai-Kizhi; BUR, Buryats; CEU, European; CHB, Han Chinese; CHK, Chukchi; E/SE Asia, East/Southeast Asia; ESK, Eskimo; EVN, Evens; EVK, Evenks; KRK, Koryaks; SHR, Shors; TUV, Tuvinians; VTN, Vietnamese; YKT, Yakuts. Reference populations are marked in italics.

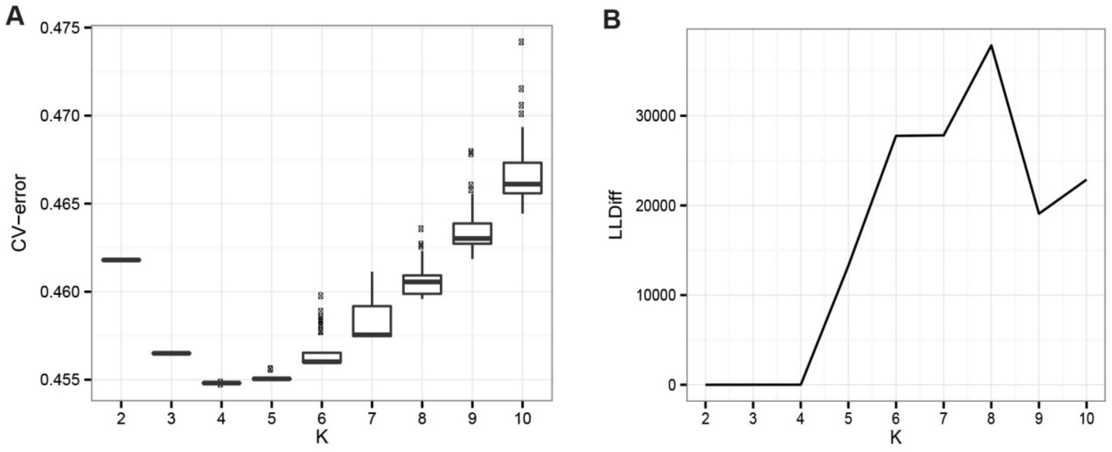

Figure S2. ADMIXTURE CV-error and log likelihood scores.
(A) CV-error scores; (B) Log-likelihood differences between the minimum log-likelihood and maximum log-likelihoods scores; for *K*=2 to 10 over the 100 iterations of ADMIXTURE analysis performed.

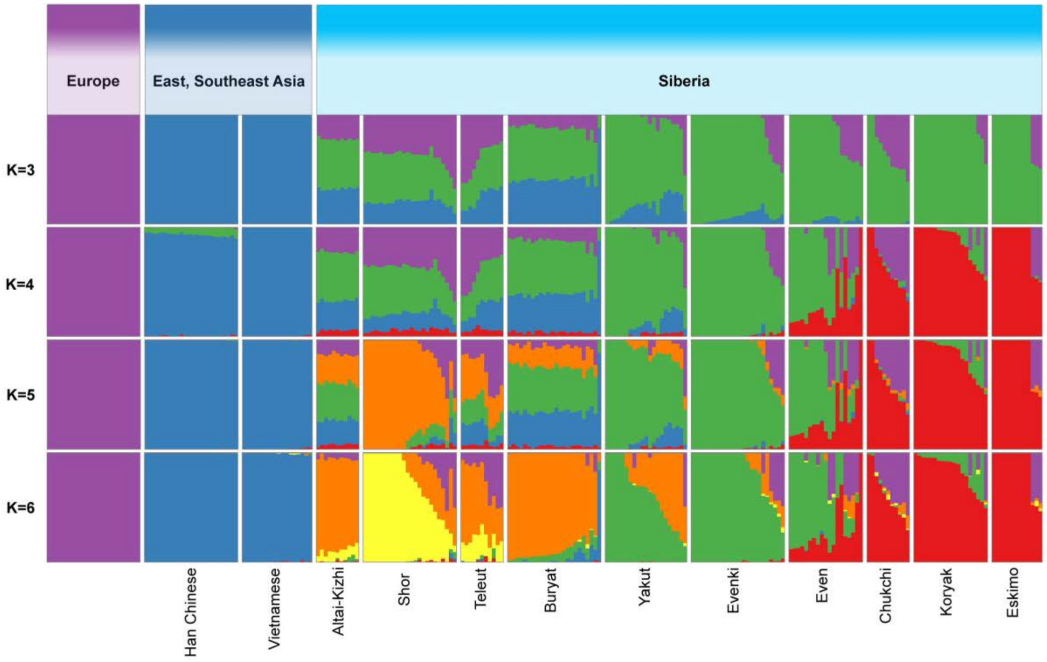

Figure S3. ADMIXTURE analysis for *K*=3 to 6 for the Siberian populations and representatives of East and West Eurasia.

ADMIXTURE plots from *K*=3 to *K*=6 of the Siberian individuals genotyped in this study together with reference populations (European, Han Chinese, Vietnamese). Each vertical line represents one sample and the different colours represent the probability of the different K ancestry that each sample is likely to be constructed from.

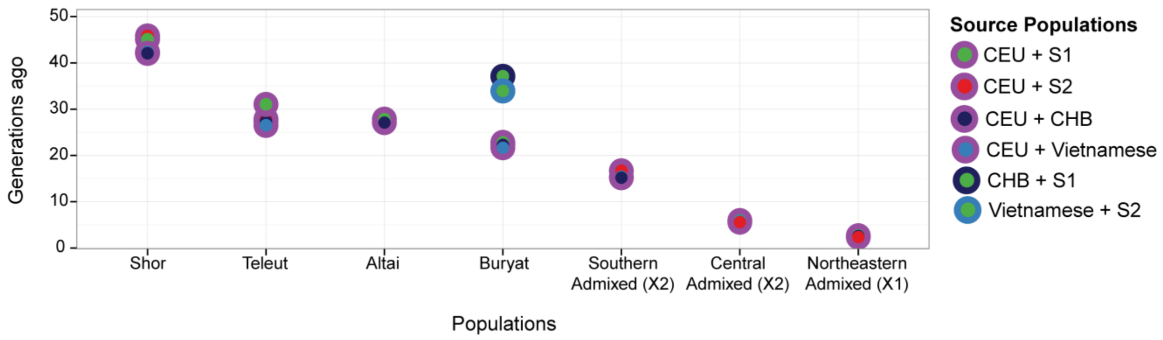

Figure S4. Admixture dating of Siberian populations and groups of admixed individuals. Each symbol shows the significant admixture dating (y-axis) for the respective population shown in the x-axis as estimated by ALDER using the source populations shown in the legend on the right hand side. "CEU" and "CHB" refer to the European and Han Chinese HapMap3 samples, "S1" refers to the unadmixed Central Siberian group and "S2" refers to the unadmixed Northeastern Siberian group (as explained in Material and Methods), "Southern Admixed (X2)" refer to the Southern Siberian admixed populations (X2), "Central Admixed (X2)" refers to the Central Siberian admixed populations (X2) and "Northeastern Admixed (X1)" refers to the Northeastern Siberian admixed populations (X1) as clustered in fineSTRUCTURE analysis (Figure 1d).

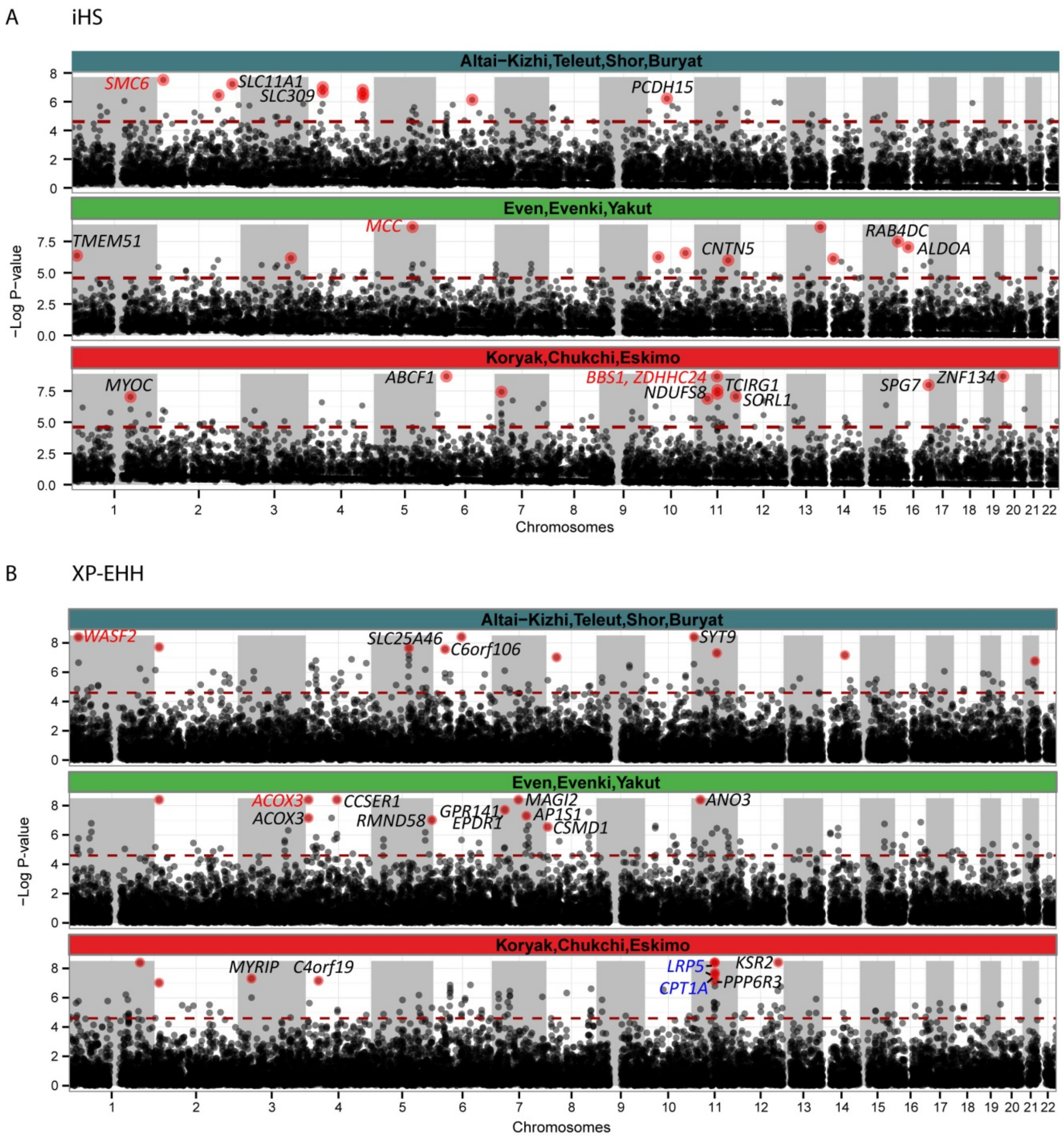

Figure S5. Genome-wide plot of empirical -$\log_{10}$ P-values of window haplotype-homozygozity scores for all Siberian groupings.

Red dashed line represents empirical top 1% threshold of the respective test. Top 10 ranking genomic windows are highlighted with red circles. Protein-coding genes present in the top 10 windows that contain the maximum |iHS| (A) and |XP-EHH| (B) scores are marked on the plot. Genes present in the topmost window of the respective population grouping are labelled in red font. Genes present in the predefined cold adaptation gene lists (Table S10) are labelled in blue font.

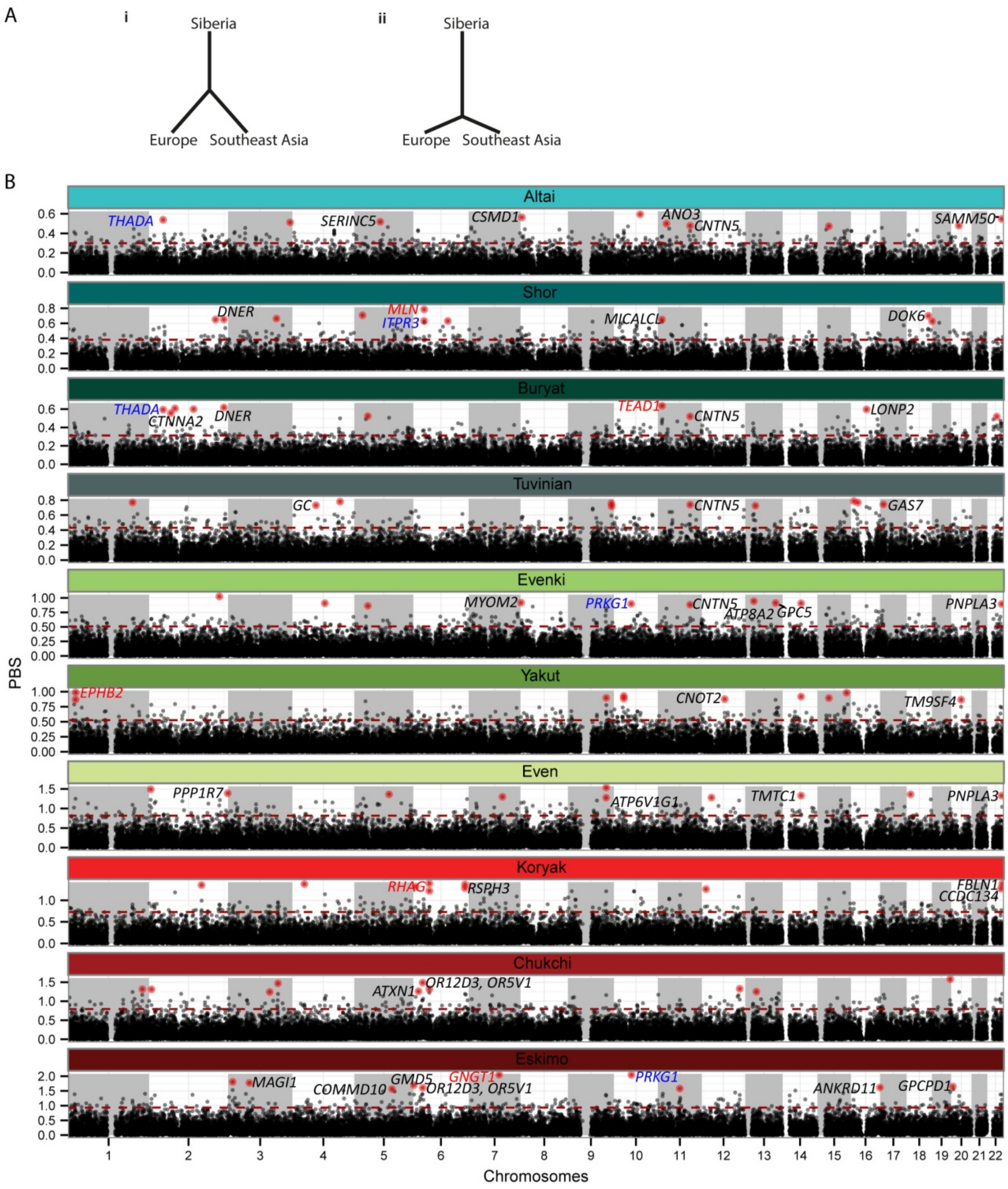

Figure S6. Genome-wide plots of maximum PBS scores in windows in the Siberian populations.

(A) PBS three-population model. i) branch lengths reflecting genome-wide average patterns of allele frequency differences; ii) high PBS scores are observed in cases of unusually high allele frequency change on the branch leading to a Siberian population. (B) Red dashed lines represents empirical top 1% threshold of the PBS tests. Top 10 ranking genomic windows are highlighted with red circles. Protein-coding genes present in the top 10 windows that contain the maximum PBS score are marked on the plots; genes present in the topmost window are labelled in red font; genes present in the predefined cold adaptation gene lists (Table S10) are labelled in blue font.

A

	ABST	EEY	CEK	VTN
ABST	1	0.398	0.186	0.354
EEY	0.468	1	0.183	0.321
CEK	0.257	0.19	1	0.124
VTN	0.409	0.282	0.209	1

B

	ABST	EEY	CEK
ABST	1	0.621	0.336
EEY	0.492	1	0.341
CEK	0.275	0.325	1

C

	Altai	Shor	Buryat	Tuvinian	Evenki	Yakut	Even	Koryak	Chukchi	Eskimo
Altai	1	0.209	0.368	0.322	0.326	0.368	0.248	0.264	0.236	0.19
Shor	0.179	1	0.187	0.195	0.183	0.187	0.144	0.202	0.128	0.128
Buryat	0.388	0.213	1	0.353	0.473	0.461	0.341	0.318	0.256	0.163
Tuvinian	0.354	0.187	0.366	1	0.374	0.362	0.335	0.296	0.23	0.191
Evenki	0.36	0.147	0.519	0.36	1	0.682	0.442	0.376	0.287	0.182
Yakut	0.36	0.174	0.422	0.345	0.64	1	0.43	0.36	0.318	0.174
Even	0.265	0.148	0.288	0.268	0.35	0.354	1	0.268	0.257	0.175
Koryak	0.198	0.174	0.264	0.283	0.326	0.341	0.357	1	0.601	0.372
Chukchi	0.19	0.14	0.209	0.236	0.279	0.271	0.329	0.589	1	0.519
Eskimo	0.143	0.12	0.147	0.159	0.205	0.236	0.178	0.333	0.453	1

Figure S7. Window sharing between Siberian groups in regions.
(A) Fraction of window sharing for iHS between Siberian groups and outgroup. A cell in the table represents the fraction of windows shared between the top 1% iHS windows of the group in the row and the top 5% iHS windows in the column respectively. (B) Fraction of window sharing for XP-EHH between Siberian groups. A cell in the table represents the fraction of windows shared between the top 1% XP-EHH windows of the group in the row and the top 5% XP-EHH windows of the group in the column respectively. (C) Fraction of window sharing for PBS between Siberian populations. A cell in the table represents the fraction of windows shared between the top 1% PBS windows of the group in the row and the top 5% PBS windows of the group in the column respectively. The following abbreviations are used in the Figure: ABST refers to the grouping of Altai-Kizhi, Buryat, Shor, Teleut populations; EEY refers to the grouping of Even, Evenki, Yakut populations; CEK refers to the grouping of Chukchi, Eskimo and Koryak populations, VTN is the reference population Vietnamese.

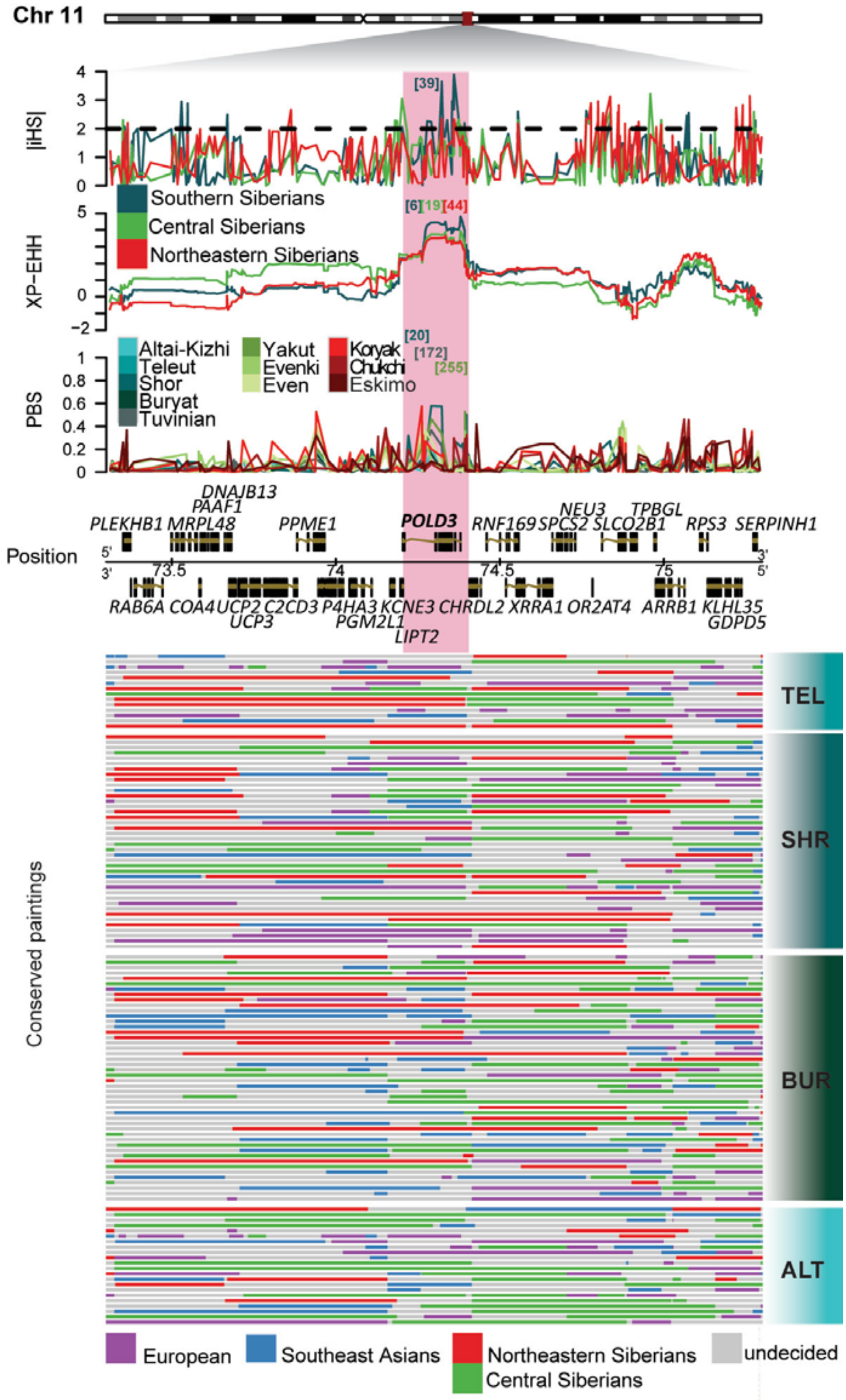

Figure S8. Zooming on the 2Mb region around the *POLD3* gene.
The |iHS|, XP-EHH and PBS scores that showed significant signals on the 2Mb region around the window that contains the *POLD3* gene are shown for the different Siberian populations. The pink highlight shows the windows present in our significant selection tests results that contain the *POLD3* gene. The position on the chromosome is given in Mb. The rankings are marked on the respective windows in the respective test panels. Protein-coding genes present in the 2Mb region are shown under the test plots. The paintings of the phased chromosomes for the region in the Southern Siberian individuals are shown underneath the *Position* legend.

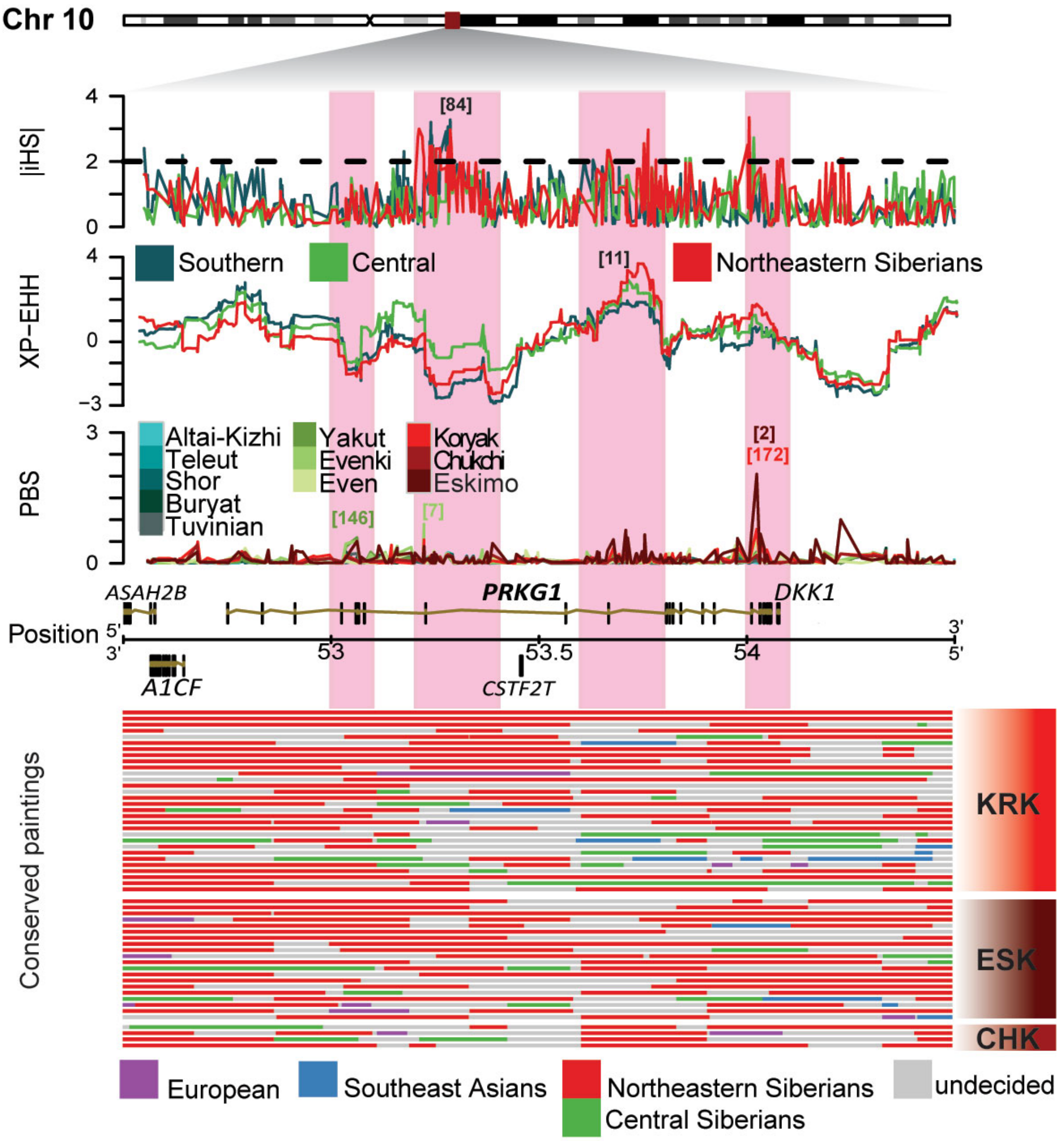

Figure S9. Zooming on the 2Mb region around the *PRKG1* gene.
The |iHS|, XP-EHH and PBS scores that showed significant signals on the 2Mb region around the window that contains the *PRKG1* gene are shown for the different Siberian populations. The pink highlights show the windows present in our significant selection tests results that contain the *PRKG1* gene. The position on the chromosome is given in Mb. The rankings are marked on the respective windows in the respective test panels. Protein-coding genes present in the 2Mb region are shown under the test plots. The paintings of the phased chromosomes for the region in the Northeastern Siberian individuals are shown underneath the *Position* legend.
